# Robust Generation of Person-Specific, Synchronously Active Neuronal Networks Using Purely Isogenic Human iPSC-3D Neural Aggregate Cultures

**DOI:** 10.3389/fnins.2019.00351

**Published:** 2019-04-24

**Authors:** Julia Izsak, Henrik Seth, Mats Andersson, Dzeneta Vizlin-Hodzic, Stephan Theiss, Eric Hanse, Hans Ågren, Keiko Funa, Sebastian Illes

**Affiliations:** ^1^Institute of Neuroscience and Physiology, Sahlgrenska Academy, Gothenburg University, Gothenburg, Sweden; ^2^Sahlgrenska Cancer Center, Institute of Biomedicine, Sahlgrenska Academy, Gothenburg University, Gothenburg, Sweden; ^3^Institute of Clinical Neuroscience and Medical Psychology, Medical Faculty, Heinrich Heine University, Düsseldorf, Germany; ^4^Result Medical GmbH, Düsseldorf, Germany; ^5^Institute of Neuroscience and Physiology, Section of Psychiatry and Neurochemistry, Sahlgrenska Academy, Gothenburg University, Gothenburg, Sweden; ^6^Oncology Laboratory, Department of Pathology, Sahlgrenska University Hospital, Gothenburg, Sweden

**Keywords:** human induced pluripotent stem cells, 3D-neural model system, neuronal networks, microelectrode array, synchronous activity

## Abstract

Reproducibly generating human induced pluripotent stem cell-based functional neuronal circuits, solely obtained from single individuals, poses particular challenges to achieve personalized and patient specific functional neuronal *in vitro* models. A hallmark of functional neuronal assemblies, synchronous neuronal activity, can be non-invasively studied by microelectrode array (MEA) technology, reliably capturing physiological and pathophysiological aspects of human brain function. In our here presented manuscript, we demonstrate a procedure to generate 3D neural aggregates comprising astrocytes, oligodendroglial cells, and neurons obtained from the same human tissue sample. Moreover, we demonstrate the robust ability of those neurons to create a highly synchronously active neuronal network within 3 weeks *in vitro*, without additionally applied astrocytes. The fusion of MEA-technology with functional neuronal circuits solely obtained from one individual’s cells represent isogenic person-specific human neuronal sensor chips that pave the way for specific personalized *in vitro* neuronal networks as well as neurological and neuropsychiatric disease modeling.

## Introduction

The human brain comprises synaptically connected neuronal networks capable of generating spontaneous synchronous electrical activity. In the absence of intracranial recording electrodes providing access to neuronal networks, in most humans neither physiological nor pathophysiological network activity can be studied *in vivo*. The introduction of induced pluripotent stem cell technology by Shinya Yamanaka (Takahashi et al., 2007) opened the possibility of generating human neurons and astrocytes from healthy and diseased individuals (Hargus et al., 2014; Heilker et al., 2014; Corti et al., 2015). Thereby, this human cell-based approach represents a promising avenue to provide insights into human neuronal electrophysiology at different functional levels. While the electrophysiological behavior of individual human iPSC-neurons has been very extensively studied, network behavior – for example the examination of the synchronous activity patterns of human iPSC neuronal cultures on a microelectrode array device (MEA) – has been challenging since solely human iPSC-derived culture systems to date have not generated networks with highly synchronous firing activity.

The formation of synchronously active neuronal networks depends on sequential developmental processes. Neuronal network formation starts with excitable and spontaneously active neurons that are asynchronously active due to the lack of functional interconnectivity between neurons. With the formation of functional synapses, two or more neurons become functionally interconnected and capable to generate synchronous bursting. In a population of neurons, the connectivity between neurons is increasing over time and finally results in synchronous bursting activity of hundreds or thousands of interconnected neurons, also referred as population bursting. While patch-clamp recordings are useful to prove excitability and synaptic function of a single neuron, calcium imaging and MEA recordings are more suitable to describe the transition of asynchronously active neurons into synchronous bursting of few neurons and finally into a population of neurons that are highly synchronously active. So far, reported MEA data sets obtained from solely human iPSC-derived neuronal populations show asynchronous neuronal firing (Kiskinis et al., 2014; Muratore et al., 2014; Wainger et al., 2014; Ho et al., 2016; Flaherty et al., 2017; Phillips et al., 2017; Xu et al., 2017). Spike raster plots with evidence for synchronous neuronal network activity have been presented (Woodard et al., 2014; Chailangkarn et al., 2016; Vessoni et al., 2016; Marchetto et al., 2017; Monzel et al., 2017). However, MEA recordings that clearly demonstrated synchronous bursting of an entire neuronal culture, referred to as neuronal population bursting, have not been included. Moreover, in some of the work calcium imaging was used, however, presented calcium traces clearly showed asynchronous calcium peaks and missed evidence for neuronal population wide synchronous network activity (Mertens et al., 2015; Chailangkarn et al., 2016; Monzel et al., 2017).

Interestingly, culturing human induced pluripotent stem cell (hiPSC)-neurons with either primary rat (Lam et al., 2017) or human astrocytes (Fukushima et al., 2016; Kuijlaars et al., 2016; Odawara et al., 2016) allows the robust generation of hiPSC-derived neuronal cultures that show population bursting detected by MEA recording devices. However, preparations of rodent astrocyte and human fetal astrocyte cultures are time-consuming and expensive procedures. Moreover, for several neurological diseases and neuropsychiatric disorders, such as bipolar disorder or schizophrenia, abnormalities in astrocytes that cause abnormal neuronal function have been speculated upon (Halassa et al., 2007; Hercher et al., 2014; Ongur et al., 2014). Thus, modeling, e.g., bipolar disorder and schizophrenia by abnormal neuronal network activity *in vitro* might require that patient-specific neurons form a functional synchronously active network within their patient-specific astroglial network. Another limiting aspect of those studies is the use of commercially available pre-differentiated hiPSC-derived neurons currently offered by several vendors. In detail, information on origin and neural differentiation of hiPSC into neurons is not disclosed and researchers become dependent on commercial vendors leading to higher costs than using in-lab produced hiPSC neural models.

Another issue is the lengthy time course of *in vitro* human neuronal circuit development necessary for generating synchronous network activity: it often takes several months (Amin et al., 2016; Odawara et al., 2016), resulting in time-consuming and expensive experiments. In contrast, rodent-cell based neural cultures give rise to functional, synchronously active, neuronal networks within less than 3 weeks (Pine, 1980; Hofmann and Bading, 2006). The different time scales of human and rodent brain development have been used to explain this temporal discrepancy between human and animal *in vitro* neuronal circuit formation. Despite the growing appreciation that the currently used culture conditions for hiPSC neural stem cell differentiation are sub-optimal (Bardy et al., 2016), the above described species-specific difference between human and rodent neuronal circuit formation has reached consensus in the neuroscience stem cell research field (Koch et al., 2009; Shi et al., 2012b; Odawara et al., 2014; Prilutsky et al., 2014; Corti et al., 2015; Livesey et al., 2015; Bardy et al., 2016; Weick, 2016). Thus, it is unclear if currently applied culture procedures do allow the robust generation of human iPSC-derived neurons on MEAs that are capable to generate highly synchronous population bursting within few days *in vitro*, without co-cultivation of astrocytes.

Here, we present highly synchronously active human neuronal networks in a purely human iPSC-based *in vitro* model without using primary rodent (Odawara et al., 2016) or human astrocyte (Kuijlaars et al., 2016) cell lines. We evaluate the efficacy of our procedure with three different hiPSC cell lines from three different healthy donors. We demonstrate that synchronous activity can be studied less than 3 weeks after seeding of human neural cells on MEAs, equal to the time described for rodent-derived neuronal cells *in vitro* (Van Pelt et al., 2004; Hofmann and Bading, 2006; Wagenaar et al., 2006). We describe the development and properties of synchronous bursting in hiPSC-derived neuronal networks, the dependency of synchronous events on neuronal excitability, synaptic glutamatergic neurotransmission and the influence of inhibitory GABAergic neurotransmission in regulating synchronous activity.

To summarize, our approach allows combining MEAs with isogenic neurons and glial cells obtained exclusively from single human individuals. MEA recordings of neuronal network activity in solely human iPSC-derived neural cells represent a promising tool for person-specific pharmaceutical drug screening and patient-specific model systems for various neurological or neuropsychiatric diseases.

## Results

### Generation of hiPSC Derived 3D-Neural Aggregate Cultures

**FIGURE 1:**
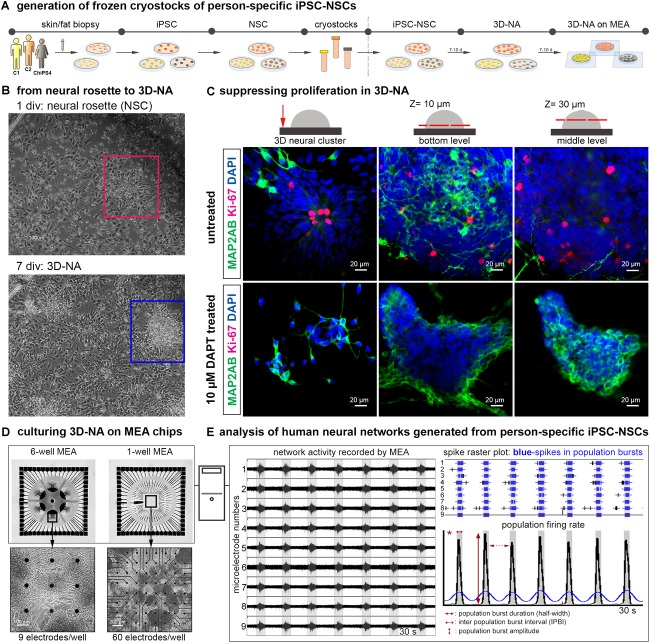
Generation and analysis of synchronously active cortical 3D-neural aggregate cultures from human iPSC cultures. **(A)** Schematic representation of the *in vitro* neural induction protocol using dual SMAD inhibition to generate cortical 3D-neural aggregate cultures. **(B)** Phase-contrast images show an overview of cell morphology in hiPSC-NSC cultures at 1 and 7 days after seeding of hiPSC-NSC cryovials. Rectangles show the transition from neural rosette (red rectangle) toward 3D-neural aggregate (blue rectangle) within 7 days *in vitro* (div). **(C)** Confocal images visualize MAP2-AB^+^-neurons and Ki-67^+^-cells in neural rosette and 3D-neural aggregates (day 30 post iPSC stage) under the indicated culture conditions. The schematic drawings illustrate the regions and *z*-levels of image acquisition. **(D)** Three different donor-derived hiPSC-NSC lines were used to generate 3D-neural aggregates seeded on 6-well (9 electrodes/well) or single well (60 electrodes/well) MEAs. **(E)** After MEA recordings, spikes were detected by an automatic spike detection algorithm and extracted from raw data by the Spanner software. Spike data were used for population bursts detection [custom-made Matlab tools as described in (Hedrich et al., 2014)].

By using the commonly applied “dual-SMAD-inhibition” protocol for neural differentiation of hiPSC (Shi et al., 2012a), we generated hiPSC-derived neural stem cells that grew as neural rosettes *in vitro*. At 20–25 days after induction of neural differentiation of hiPSC, hiPSC-NSC cultures could be stored as cryostocks at −152°C for further experiments (see schematic description of used procedures in Figure 1A). After thawing and seeding of hiPSC-NSC, dissociated NSC reorganized into neural rosettes within 1–2 days, which further developed into 3D-neural aggregates (Figure 1B), as reported by others (Edri et al., 2015). These morphological analyses indicate on-going proliferation of NSC, which was further confirmed by immunocytochemistry staining and confocal imaging of Ki67^+^ cells (Figure 1C). In detail, Ki67^+^ cells were localized within the apical side of neural rosettes and throughout 3D neural aggregates (Figure 1C, untreated). Since inhibition of gamma-secretase with N-[(3,5-difluorophenyl)acetyl]-L-alanyl-2-phenyl]glycine-1,1-dimethylethyl ester (DAPT) enhances neuronal differentiation and suppresses proliferation in human iPSC neural cultures (Borghese et al., 2010; Kirkeby et al., 2012), we used DAPT to obtain hiPSC-derived neural cultures showing absence of Ki67^+^ cells and numerous MAP-2AB^+^ neurons (Figure 1B, DAPT-treated).

Our previous work showed that a 3D neural environment enhances functional neuronal circuit formation of mouse embryonic stem cell-derived NSC (Illes et al., 2009). Thus, we transferred hiPSC 3D-neural aggregate cultures to MEAs in order to assess the development of neuronal network activity (Figure 1). We used a cultivation media, BrainPhys^TM^, with a physiological salt concentration similar to cerebrospinal fluid in order to create a more physiological environment (Bardy et al., 2015), representing a further major difference from previous studies (Heikkila et al., 2009; Kirwan et al., 2015; Fukushima et al., 2016; Kemp et al., 2016). To further prevent neural overgrowth and enhancement of neuronal differentiation, we supplemented the cultivation media with neurotrophic factors (NT-3, FGF18, BDNF, GNDF, TGF-β, DAPT). The described procedures were evaluated on hiPSC-NSC obtained from three different healthy individuals [two males – C1, C2 see (Vizlin-Hodzic et al., 2017), and one female (ChiPSC4, Cellartis), culturing them on MEAs (Figure 1)]. By this, we achieved robust and long-term attachment of 3D neural aggregates on MEA recording arrays over several weeks (Supplementary Figure S1).

To describe the development and properties of neuronal network activity, we used 6-well MEAs (six separated wells with nine electrodes each) as well as 1-well MEA chips, which are composed of a single well with 59 recording electrodes and one reference electrode (Figure 1D). Every 2–4 days, we performed MEA recordings to determine at which time point synchronous neuronal activity appeared. In addition, phase-contrast imaging was used to identify the morphological properties of 3D-neural aggregates and to identify the localisation of synchronously active neurons in 3D-neural aggregate cultures. We used the SPANNER software for spike detection and applied a custom-made MATLAB tool to characterize number of spikes, Cohen’s kappa (measure of spike synchrony), population burst firing in terms of number and inter-event interval of population bursts [Figure 1E, for more details see method section and (Hedrich et al., 2014)]. Importantly, population bursts detected by using our custom-made MATLAB tool were compared to the MEA-recordings to exclude the detection and analysis of false-positive population bursts.

### Synchronously Active Neuronal Networks Within 3 Weeks by Using Solely Person-Specific Human iPSC Derived Neural Cells

**FIGURE 2:**
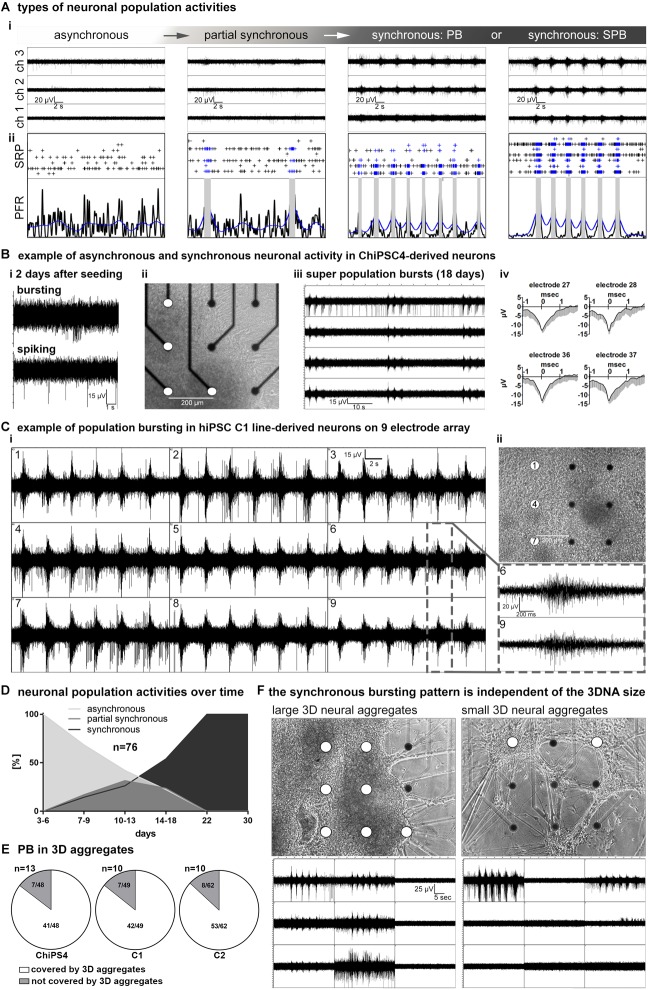
network maturation of hiPSC derived 3D-neural aggregate cultures. **(A,i)** Exemplary images of MEA-recordings with **(A,ii)** corresponding spike raster plots (SRP) and population firing rate (PFR) diagrams illustrate the different stages of neuronal network maturation from asynchronously active to highly synchronously active neuronal population activity. **(B,i)** Example of uncorrelated spiking and bursting activity, **(B,ii)** phase-contrast image, **(B,iii)** MEA-recording of synchronous population bursting, **(B,iv)** mean spike shapes detected by electrodes (white circles) in ChiPS4-derived 3D-neural aggregates placed on nine electrodes of a 6-well MEA. **(C,i)** Synchronous population bursting detected by electrodes (white circles in **ii**) covered by hiPSC-C1- derived 3D-neural aggregates and **(C,ii)** corresponding phase-contrast image 16 days after seeding on a 6-well MEA. Gray box shows details of recorded population bursts depicted from **B,i**. **(D)** Diagram summarizes the percentage of 76 individual neuronal cultures showing asynchronous, partially synchronous or synchronous activity at indicated time points. **(E)** Diagrams show that electrodes which are detecting population bursting (PB) are predominantly covered by 3D neural aggregates. **(F)** Phase-contrast images show that neuronal cultures comprised of 3D neural aggregates with different diameters show identical pattern of population bursting visualized by corresponding images of MEA recordings. Note, both cultures are derived from the same human iPSC line, experiment and MEA recording was performed at the same time point.

The development of human iPSC-neuronal network activity *in vitro* can be described as three sequential stages: (a) asynchronous, (b) partially synchronous and (c) highly synchronous neuronal population activity stage (Figure 2A). MEA recordings performed 1–2 days after seeding of 3D-neural aggregates on MEA chips revealed uncorrelated spontaneous spiking (electrodes detecting more than 10 spikes/minute are considered as active electrodes) and bursting (that is >3 spikes per 50 ms) activity (Figure 2A,B(i)). At this time point, no synchronous bursting activity of neurons appears, and we thus termed this neuronal population activity as asynchronous, which indicates that not all neurons were functionally interconnected. The recorded signals showed typical shape and duration of extracellularly recorded neuronal action potentials (spikes) (Figure 2B(iv)). These demonstrate that neurons within the 3D-neural aggregates were electrophysiologically active. Within additional 2–3 weeks, we recorded highly synchronous bursting detected by spatially distributed electrodes (Figure 2A,B(iii),C). We observed that highly synchronously active cultures showed either single individual population bursts (PB, Figure 2A,C) or super-population bursts (SPB, Figure 2A,B(iii)), which comprise a sequence of population bursts followed by a network refractory period, in which neuronal activity is strongly reduced or even absent. Inbetween the asynchronous and the synchronous activity stage, we observed that hiPSC-3D neural aggregate cultures showed partially synchronous population activity, which is characterized by few population bursts randomly appearing in between uncorrelated asynchronous neuronal activity (Figure 2A).

To describe the time line of the transition from asynchronous and partially synchronous into highly synchronous activity neuronal networks, we performed a qualitative assessment of MEA-recordings, spike raster plots and population firing diagrams from 76 individual neural cultures obtained from three different iPSC lines. We defined six time windows and evaluated the percentage of hiPSC-3D neural aggregate cultures showing asynchronous, partially synchronous and highly synchronous (population bursting and super-population bursting) at a specific time period (Figure 2D). During the first days (3–6 days) after seeding of 3D-neural aggregates on MEAs, only asynchronous network activity could be detected (Figure 2D). Partially synchronous network activity appeared between 7 and 22 days (Figure 2D). After 22 days all cultures were synchronously active showing either several individual rhythmic population bursts or super population bursts (Figure 2D).

Next, we analyzed if electrodes covered by 3D-neural aggregates or cells that grew out from 3D-neural aggregates detected synchronous bursting. For this analysis we used 33 individual neuronal cultures obtained from three different hiPSC lines (Figure 2E). We observed that 85,5 ± 0,1% of recorded PBs were detected in 3D neural aggregates and only 13,9 ± 0,9% of recorded PBs were detected outside of 3D neural aggregates (*n* = 33, mean ± SD, *p* < 0,0001, see also Figure 2E). Electrodes that were not covered by 3D-neural aggregates or cells did not show neuronal activity. This data shows that synchronously active neurons are predominantly located within 3D-neural aggregates.

We observed that 3D neural aggregates varied in size, which indicates that the numbers of cells within 3D neural aggregates varied between different cultures. Interestingly, we observed that the patterns of population bursting recorded from “large” (Figure 2F, left) and “small” (Figure 2F, right) 3D-neural aggregates at the same time were identical (Figure 2F, compare left and right MEA recording image). This shows that the pattern of synchronous neuronal activity generated by neurons within 3D-neural aggregates is independent of the cell number within 3D-neural aggregates.

**FIGURE 3:**
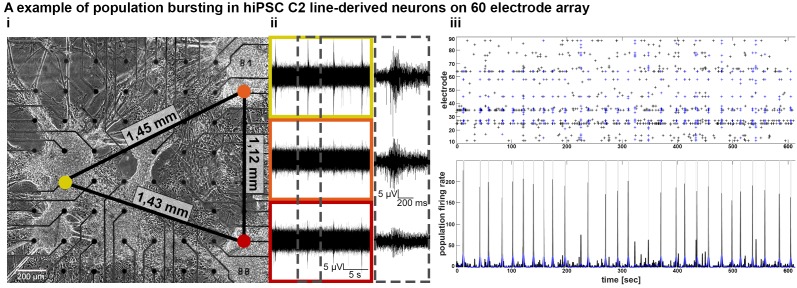
Establishment of functional connectivity and population bursting of spatially separated 3D neural aggregates within 3 weeks *in vitro*. **(A,i)** Phase-contrast image and **(A,ii)** synchronous population bursting detected by electrodes [colored circles in **(A,i)** are equivalent to boxes in **(A,ii)**] covered by spatially distributed hiPSC-C2-derived 3D-neural aggregates at 18 days after seeding on a 60-electrode MEA. Numbers at black lines indicate the distance in mm between the marked electrodes and spatially separated 3D-neural aggregates. Gray box shows details of recorded population bursts. **(A,iii)** Spike raster plot and population firing rate visualize the synchronous populations bursting from culture shown in **(A,i)**.

To confirm that neurons located in spatially separated 3D neural aggregates were functionally interconnected, we cultured 3D neural aggregates on 60-electrode MEAs that have a larger recording area than 9-electrode MEAs. We observed synchronous bursting activity in spatially distributed (more than 1,4 mm distance) 3D-neural aggregates after 14 days of cultivation (Figure 3). These data demonstrate that neuronal populations within spatially distributed 3D neural aggregates establish functional interconnections within 2 weeks of cultivation.

To summarize, neurons within 3D neural aggregates form functional synchronously active neuronal networks nearly at identical time points, independent of which human iPSC line is used and independent of 3D-neural aggregate size.

### Quantitative Assessment of Neuronal Network Development

**FIGURE 4:**
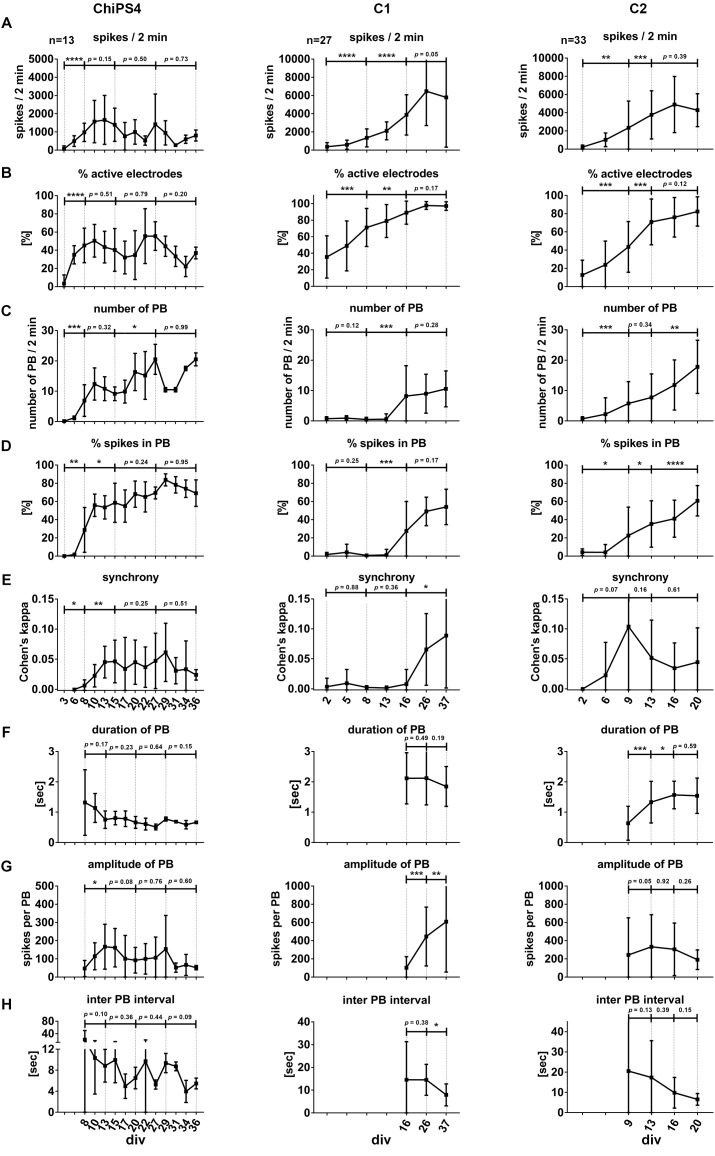
Neuronal network development and properties of synchronous firing in iPSC-derived human neuronal networks. Bar diagrams show the temporal change of neuronal network parameters recorded from several individual neural cultures (indicated as *n* = number of individual cultures per cell line) obtained from ChipSC4, C1 and C2 human iPSC lines (columns), **A–H** mark the different network parameters from the three different cell lines. Values are shown as mean ± SD. Asterisks indicate significant values (^∗^*p* < 0.05; ^∗∗^*p* < 0.01; ^∗∗∗^*p* < 0.001, and ^∗∗∗∗^*p* < 0.0001). For details see main text.

For a quantitative and comparative assessment of the functional development of neuronal networks, we analyzed different neuronal network parameters of 76 individual neural cultures obtained from three different hiPSC-lines (Figure 4). The parameter “spikes per minutes” provides information about the overall activity of neurons; however, this value depends on the number of electrodes detecting neuronal activity (% of active electrodes). All neuronal cultures showed a developmental phase in which the number of recorded spikes (Figure 4A) and the percentage of active electrodes (Figure 4B) increased over time and reached a plateau phase. However, the time line of this developmental phase differed between the neuronal populations obtained from different cell lines.

To describe the formation of a synchronously active neuronal network, we used the parameters “number of population bursts” (number of PB, Figure 4C), “percentage of spikes organized in population bursts” (% spikes in PB, Figure 4D) and “Cohen’s kappa” to describe synchrony of neuronal activity (synchrony, Figure 4E). The time point when the average value of detected PB exceeded the value of five was different between the three hiPSC-derived 3D neural aggregate cultures (for neuronal populations derived from ChiPSC4: 8 days, C1-derived neuronal population: 16 days, C2-derived neuronal population: 9 days).

In ChiPSC4-derived neuronal populations, we observed that the average number of detected PB increased every week and was 20 ± 4.9 at day 27. A reduced number of PB appeared between days 29 and 34; however, at day 36 the average number of PB turned back 20 ± 2.1 PB. The % of spikes organized in PB increased from 28.7 ± 24.5% at day 8 to 69.1 ± 14.5% at day 36. The plot for synchrony shows that the Cohen’s kappa value increased from 0 (day 6) to 0.045 ± 0.02 (day 13) and did not show significant change up to day 36.

In C1-derived neuronal populations, the average number of detected PB remained unchanged between day 16 (PB = 8.2 ± 9.9) and day 37 (PB = 10.5 ± 5.9). The % of spikes organized in PB increased from 27.6 ± 32.4% at day 16 to 54.0 ± 19.4% at day 37, however, this increase was not significant. The plot for synchrony shows that the Cohen’s kappa value increased from 0 (day 16) to 0.088 ± 0.087 (day 37).

In C2-derived neuronal populations, the average number of detected PB increased on weekly basis and was 17.8 ± 8.7 at day 20. The % of spikes organized in PB increased from 22.6 ± 31.1% at day 9 to 60.7 ± 16.6% at day 20. The plot for synchrony shows that the Cohen’s kappa value increased from 0 (day 2) to 0.1 ± 0.1 (day 9) and reduced to 0.05 ± 0.05 at day 20. However, these changes were not statistically significant.

Next, we used duration (i.e., half width of peak amplitude) and amplitude (i.e., spikes per population burst) of population bursts to describe the properties of synchronous bursting over time.

In ChiPSC4-derived neuronal populations, the average duration of population bursts started with 1.3 ± 1.1 sec at day 8 and weakly reduced to 0.7 ± 0.05 s at day 36. The PB amplitude increased from 50 ± 47 spikes/s per PB at day 8 to 180 ± 123 spikes/s per PB at day 3. For 2 weeks, the amplitude of PBs did not significantly change, however, it showed a reduction between 31 and 36 days.

In C1-derived neuronal populations, the average duration of population bursts started with 2.1 ± 1.2 s at day 16 and did not significantly change till day 37. The PB amplitude increased weekly from 90 ± 93 spikes/s per PB at day 16 to 600 ± 560 spikes/s per PB at day 37.

In C2-derived neuronal populations, the average duration of population bursts started with 0.6 ± 0.5 s at day 9 and increased to 1.5 ± 0.4 s at day 16, and did not significantly change till day 20. The PB amplitude PBs remained unaltered during this time period (day 9: 220 ± 400 spikes/s per PB, day 20: 200 ± 100 spikes/s per PB).

In line with the increased number of PB over time, the inter-PB interval decreased in all neuronal cultures obtained from three different hiPSC lines (Figure 4H). At early time points in culture, the inter-PB interval was tens of seconds long in all three hiPSC-line derived 3D neural aggregate cultures. However, at the latest time points in culture, the inter-PB interval was below 10 s in all three hiPSC-line derived 3D neural aggregate cultures.

### Conserved Pattern of Population Bursting Among Individual 3D Neural Aggregate Cultures

**FIGURE 5:**
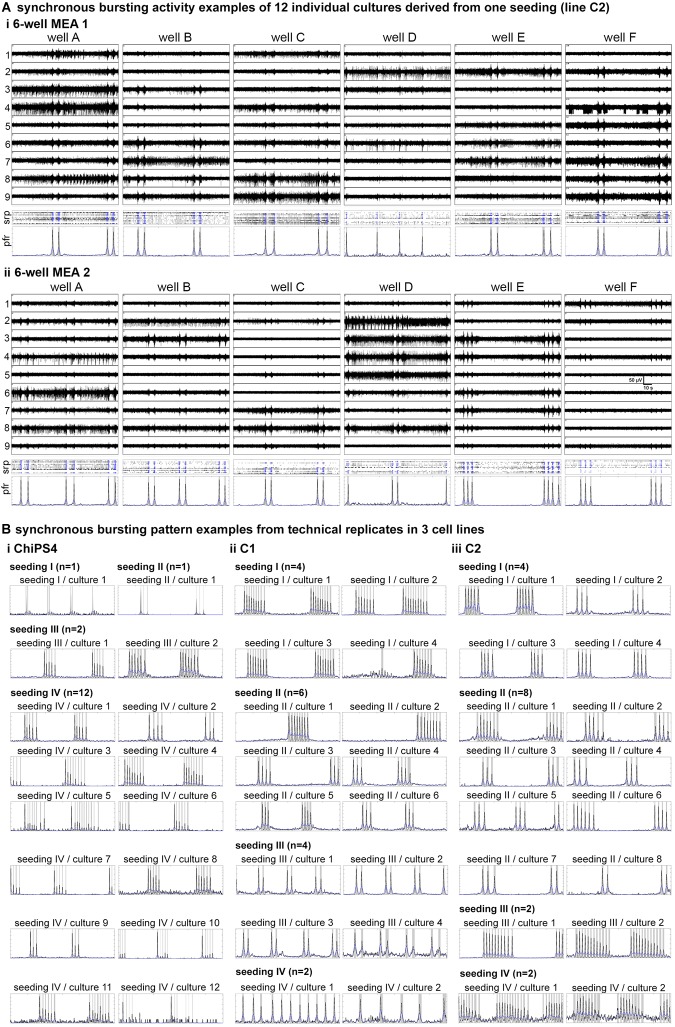
Conserved pattern of population bursting among individual 3D neural aggregate cultures. **(A)** MEA recordings, spike raster plots (SRP) and population firing rate (PFR) diagrams of C2-derived 3D-neural aggregate cultures (*n* = 12) 10 days after plating on 6-well MEAs are showing low inter-culture variabilities in synchronous bursting pattern. The channel numbers are marked at the left side of the representative MEA recordings. Crosses in SRP diagrams indicate detected spikes, where blue crosses stand for spikes detected within population bursts (PB). PBs are marked with gray vertical bars in PFR diagrams. For neuronal network parameters see also Supplementary Table S1 **(B)** Population firing diagrams showing the pattern of population bursting (gray vertical bars) recorded from **(B,i)** ChiPS4, **(B,ii)** C1 and **(B,iii)** C2-derived 3D neural aggregates on 6-well MEAs from four different seedings. Each seeding represents an experiment in which different batches of human iPSC-NSC cryovials (four batches per cell line) were treated as shown in Figure 1 to generate 3D neural aggregates on MEAs. *N* indicates the number of individual cultures per cell line.

By comparing MEA recordings, spike raster plots and population firing rate diagrams measured at the same time, we observed that the pattern of synchronous activity was very similar or even identical among individual 3D neural aggregate cultures (Figure 5A). For instance, MEA recordings performed in twelve 3D-neural aggregate cultures growing in two different 6-well MEAs at the same time point showed either identical or very similar population burst patterns (Figure 5A). In detail, nine out of twelve 3D-neural aggregate cultures showed rhythmic population bursts with identical patterns, i.e., 2 PB per SPB (Figure 5A). These 3D-neural aggregate cultures had a PB duration of 1.51 ± 0.28 s (%CV = 18%) and an inter-PB interval of 20.5 ± 0.28 s (%CV = 21%). Similar to this, 3D neural aggregate cultures with 1 PB (1 out 12) or 3 PB per SPB (2 out of 12) had a PB duration of 1.06 ± 0.43 s and an inter-PB interval of 21.5 ± 2.65 s. Other population burst parameters, e.g., amplitude and Cohen’s kappa, had relative coefficient of variation ≥40%, and thus, are not suitable to numerically describe the similarities between different neuronal networks (see Supplementary Table S1). We compared population bursting patterns recorded from 3D neural aggregates that were obtained from 11 different batches of cortical differentiation of three different human iPSC lines (Figure 5B). We observed (i) that all neuronal populations obtained from 11 different batches formed synchronously active neuronal networks, (ii) that neuronal networks obtained from different seedings (experiments) showed differences in their patterns of population bursting, and (iii) that patterns of population bursting in neuronal cultures from the same seeding (experiment) were very similar or identical (Figure 5B).

In summary, these data demonstrate the robustness of our procedure to generate highly synchronously active human neuronal circuits. We demonstrate that our method can be applied on different batches of human iPSC-NSC obtained from different human iPSC lines and neural differentiations to achieve synchronous human neuronal networks, which shows very low well-to-well variability in the pattern of synchronous neuronal network activity.

### Human iPSC-Derived 3D-Neural Aggregates Are Composed of Synaptically Interconnected Cortical Neurons Surrounded by Endogenously Developed Human Glial Cells

**FIGURE 6:**
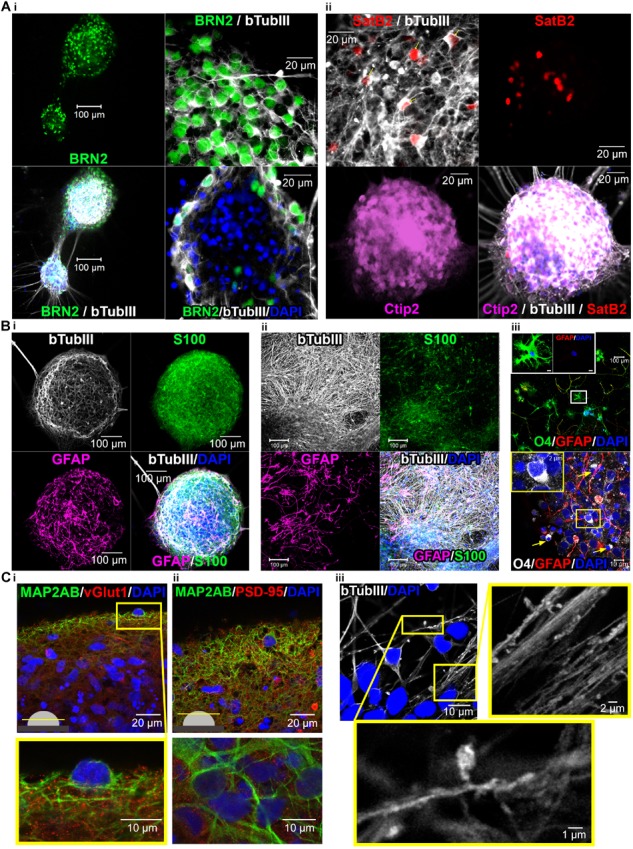
Confocal images show cortical neuronal identity, presence of glial cells, synapses and spines in 3D-neural aggregate cultures. **(A,i)** BRN2+, **(A,ii)** CTIP2+, and SATB2+ cortical βTubIII+-neurons within 3D-neural aggregates. **(B,i,ii)** βTubIII^+^-postmitotic neurons were embedded in a glial network comprising GFAP^+^, S100β^+^ astrocytes and **(B,iii)** O4^+^ oligodendrocytes present outside (above) and at the edges (below, arrows) of 3D-neural aggregates. Boxes show morphology of O4^+^ oligodendrocytes at higher magnification. **(C,i,ii)** Visualization of excitatory vGlut^+^ and PSD-95^+^ synapses as well as **(C,iii)** βTubIII^+^-filled spines within 3D-neural aggregates. All images were taken 28 days after neural aggregate isolation and cultivation of ChiPSC4-derived neural cultures.

**FIGURE 7:**
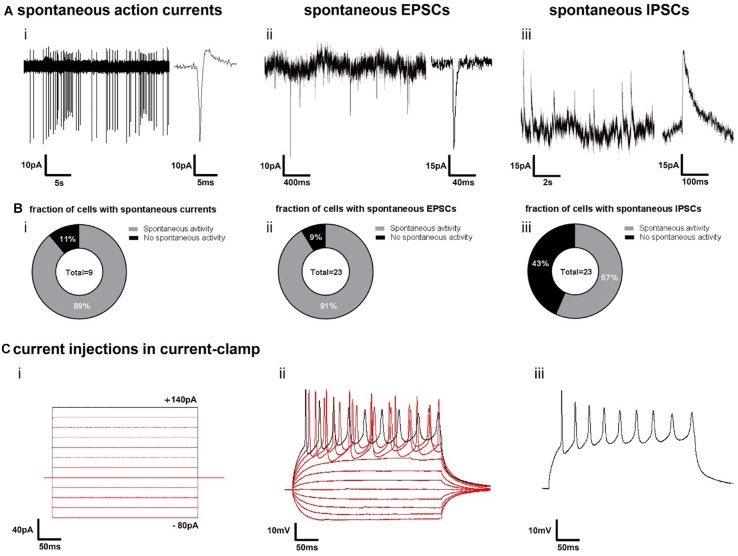
Electrophysiological function of neurons in 3D-neural aggregates on synapse and single cell level. **(A)** Examples of cell-attached recordings of **(i)** spontaneous action currents, whole-cell patch-clamp recordings of **(ii)** spontaneous excitatory post-synaptic currents (EPSCs) and whole-cell patch-clamp recordings of **(iii)** spontaneous inhibitory post-synaptic currents (IPSCs) at the edges of 3D neural aggregates (14–21 days *in vitro*) **(B)** Diagrams show the percentage of nine patched cells with **(i)** spontaneous action currents and 23 patched cells with **(ii)** EPSCs and **(iii)** IPSCs. Experiments were conducted between the second and third week of 3D neural aggregate cultivation. **(C)** Example of evoked firing responses to several 300 ms stepwise increasing current injections **(i)** into a current-clamped neuron **(ii)**, showing also the isolated firing response to a single current injection **(iii)**.

In order to obtain insights into the cellular composition of synchronously active neural circuits, we cultivated 3D-neural aggregates followed by immunocytochemistry and confocal imaging after 28 days of cultivation — at a time point when synchronously active neuronal networks were established. 3D-neural aggregates contained early-born layer V CTIP2+, middle-born II/III/V-BRN2, and few late-born layer II/III-SATB2+ cortical βTubIII+ neurons (Figure 6A). A further hallmark of mature cortical circuits was the presence of a glial network created by mature astrocytes. We observed that GFAP+/S100β+ and GFAP–/S100β+ astrocytes were organized as a glial network throughout the entire 3D-neural aggregates (Figure 6B(i)). Outside of the 3D-neural aggregates, βTubIII+-neurons grew on top of the GFAP+/S100β+ and GFAP-/S100β+ astrocytic network (Figure 6B(ii)). We also identified O4+/GFAP+ and O4+/GFAP-oligodendroglial cells within and outside of 3D-neural aggregates (Figure 6B(iii)), demonstrating that oligodendroglia development occurred within 28 days of neural differentiation. Optical slices (0.5 μm) taken at the middle and at the top of the neuronal network revealed a very dense distribution of excitatory vGlut1+ (Figure 6C(i)) and PSD-95+ synapses (Figure 6C(ii)) appearing as dot-like structures along MAP2-AB+ neuronal processes within 3D-neural aggregates (Figure 6C). Note that vGlut1+ and PSD-95+ positive structures always had a dotted appearance, and we did not observe a continuous cytoplasmic distribution of these synaptic proteins as has been reported for immature neurons (Illes et al., 2009). In addition, we observed several neurons showing βTubIII+ dendritic spines along single or a bundle of βTubIII+ neurites (Figure 6C(iii)). Complementary cell-attached and whole cell patch clamp recordings demonstrate that neurons at the edges of 3D neural aggregates were excitable, spontaneously active and showed spontaneous excitatory (ePSCs) and inhibitory post-synaptic currents (iPSCs) (Figure 7). In detail, we observed that 8 out of 9 neurons showed spontaneous neuronal activity (Figure 7B(i)), which were measured as spontaneous action currents in cell-attached configuration (Figure 7A(i)). We observed that most neurons showed ePSCs (20 out of 23 neurons, Figure 7A(ii),B(ii)) and fewer neurons showed iPSCs (13 out 23 neurons, Figure 7A(iii),B(iii)), which relies on the cortical identity of neurons. Note, that all 23 patched neurons showed evoked response after current injections (Figure 7C). Complementing the electrophysiological data, visualization of mature synaptic proteins and spines within human cortical neurons embedded in a human glial network further demonstrated that 3D-neural aggregates comprised highly functional neuronal cortical circuits.

### Synchronous Activity Depends on Neuronal Excitability, Glutamatergic, and GABAergic Synaptic Transmission

**FIGURE 8:**
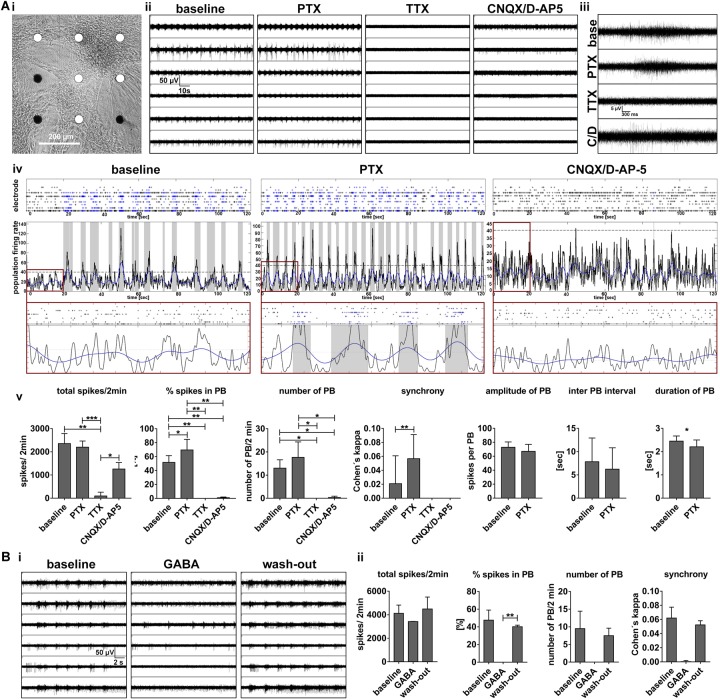
Synchronous population bursting depends on neuronal excitability and is regulated by glutamatergic and GABAergic neurotransmitter systems. **(A,i)** Phase-contrast image of 3D-neural aggregates cultured on nine electrode MEA to visualize the position of electrodes detecting synchronous population bursting (white electrodes) **(A,ii)** Synchronous population bursting recording before (baseline) and after the application of picrotoxin (PTX, 50 μM), tetrodotoxin (TTX, 500 nM), CNQX, D-AP5 (50 μM, each). **(A,iii)** Example of population bursts detected by a particular electrode before and after compound application. **(A,iv)** Spike raster plots (SRP) and population firing rate diagrams (PFD) illustrate changes in population bursting (blue spikes in SRPs and gray vertical bars in PFD before and after inhibition of GABA_A_-receptors (application of Picrotoxin) and inhibition of fast glutamatergic synaptic transmission (application of CNQX, D-AP5). Red boxes show the SRP and PFR with the same *x* and *y* scale for the different conditions (*x*: 20, *y*: 45). **(A,v)** Bar diagrams show the temporal change of neuronal network parameters recorded before and after the application of compounds (*n* = 6, mean ± SD). **(B,i)** MEA recording before (baseline), after the application of GABA (5 μM) and the following washout. **(B,ii)** Bar diagrams show the temporal change of neuronal network parameters recorded before and after the application of GABA as well as washout (*n* = 2, mean ± SD). Asterisks indicate significant values (^∗^*p* < 0.05, ^∗∗^*p* < 0.01, ^∗∗∗^*p* < 0.001, and ^∗∗∗∗^*p* < 0.0001).

To further validate the functional contribution of glutamatergic and GABAergic neurons in regulating population bursting activity, we performed pharmacological experiments. Disinhibition-mediated increased population bursting produced by GABA_A_ receptor blockage is a well-characterized feature of *in vitro* neuronal networks (Streit et al., 2001; Illes et al., 2007, 2014). Blocking of GABA_A_-receptors with picrotoxin [50 μM] resulted in an increase of synchronous network events in hiPSC-derived 3D neural aggregate cultures (Figure 8A), which represents a supplementary proof of functionality of the cultured neuronal network. Complementary, population bursting was absent after blocking the NMDA and AMPA receptors by D-AP5 and CNQX, respectively (50 μM, each, Figure 8A) or activating GABA-receptors by GABA (Figure 8B). However, residual asynchronous spiking and bursting could be observed in the absence of fast synaptic glutamatergic or activation of GABAergic transmission, most likely generated by intrinsically active neurons, as we described previously for mouse pluripotent stem cell-derived neuronal circuits (Illes et al., 2014). However, all neuronal activity was completely absent after blocking of voltage-gated sodium channels with TTX [500 nM], demonstrating that all recorded activity solely depended on neuronal excitability (Figure 8). To summarize, the here presented hiPSC-neuronal network activity was regulated by the excitatory function of glutamatergic neurons, the inhibitory function of GABAergic neurons and depended on neuronal excitability.

## Discussion

By using several neural cultures from three different hiPSC lines, we present the robust, reproducible and fast generation of *in vitro* human neuronal networks with highly synchronous firing activity of purely human origin. We combined the two powerful MEA and human iPSC technologies to demonstrate that human neurons within hiPSC-derived 3D-neural aggregates developed to functional neuronal networks within less than 3 weeks without any application of rat or human astrocytes, astrocyte-conditioned media or any other animal-based components (Fukushima et al., 2016; Odawara et al., 2016). The presented functional 3D human neural model comprises human neurons, astrocytes and oligodendroglial cells generated solely from hiPSC originating from the same individual, which represents a prerequisite for person-specific drug screening and CNS disease modeling. Moreover, we demonstrate that this human brain cell-based platform permits the evaluation of pharmacological testing in human neuronal networks.

In the following, we discuss our findings in the context of recent progresses in the field of stem cell research, human neural circuit development, and translational medical applications.

### Comparison to Previous Approaches to Achieve Highly Synchronously Active Human iPSC-Based Neuronal Circuits *in vitro*

Within recent years, combining hiPSC-derived neurons with MEA technology has become increasingly attractive. However, this has been challenging since solely human iPSC-derived functional neuronal circuits, which contain only isogenic glial cells, have not generated robust networks with highly synchronous firing activity to date. The major challenges are (i) to create *in vitro* conditions that promote neuronal maturation and circuit development of human neurons without using xenogenic or allogenic added astrocytes, (ii) to ensure that neurons remain in close vicinity to the extracellular recording electrodes by maintaining the attachment of neurons on the electrode array surface, (iii) to apply algorithms for the detection of synchronous neuronal activity, and (iv) to avoid spurious detection of synchronous bursts in MEA data that contain also uncorrelated spike and burst activity.

To obtain synchronously active hiPSC-neurons within a short time period, we used *in vitro* conditions in which maturation of human neurons occurred in a 3D isogenic cellular environment. We used BrainPhys media, which supports neuronal electrophysiological function, in contrast to neurobasal or DMEM-based culture media (Bardy et al., 2015). We also did not supplement our culture media with bovine serum as has been reported (Odawara et al., 2016), because a previous study showed that serum reduced the activity of human neurons (Bardy et al., 2015; Odawara et al., 2016). We did not use rat astrocytes or commercially available human iPSC-derived astrocytes, because our presented approach does allow the simultaneous generation of neurons, astrocytes and oligodendrocytes by using human iPSC obtained from the same individual. In previous studies, synchronously active human iPSC-derived neurons were obtained by co-culture approaches with rat (Odawara et al., 2016) or human iPSC-derived astrocytes (Tukker et al., 2018). For example, Tukker et al. (2018) purchased human iPSC-derived neurons and human iPSC-derived astrocytes from Cellular Dynamics International (Madison, WI, United States), to achieve synchronously active neurons. Since the origin of used cells is unknown and the neural differentiation procedure has not been fully disclosed, it is unclear if neurons and astrocytes were obtained from the same human iPSC line (isogenic model) or from different human iPSC lines (allogenic model). In general, we consider the use of commercially available neurons and astrocytes is limiting scientific research potential, because research becomes dependent on the availability of expensive cells, while important information about cell origin and neural differentiation procedures are not disclosed. For instance, one forte of human iPSC-technology is given by the ability to study and modulate iPSC stage neural development to understand how different neural stem cell populations form functional neuronal circuits *in vitro*. Moreover, iPSC technology does allow the generation of person-specific *in vitro* models, in which all cells were derived from the same individual, and thereby, all *in vitro* cells have the identical genetic background. Thus, we describe for the first time the robust and fast generation of a person specific functional neuronal model system, in which synchronously active hiPSC-neurons, astrocytes, and oligodendrocytes were generated from the same human iPSC line.

Detection and analysis of synchronous neuronal activity represent a general challenge in neuroscience (Cotterill and Eglen, 2018). To prove that detected population bursts truly reflected synchronous neuronal activity detected by several spatially distributed electrodes, we carefully confirmed the detection of population bursts by comparing MEA recordings with their corresponding spike raster plots and population firing rate diagrams (as described in Figure 1). This is particularly crucial for partially synchronously active neuronal networks showing sparse population bursting. In addition, we demonstrate that synchronous bursting is absent after pharmacological inhibition of neuronal excitability, inhibition of excitatory glutamatergic synaptic communication, or activation of inhibitory GABAergic synaptic transmission. *Vice versa*, we demonstrate that synchronous activity can be increased by inhibition of inhibitory GABAergic synaptic communication. Several studies that aim at generating synchronously active neuronal networks from hiPSC neurons do not include images of MEA recordings and present only few representative examples of spike raster plots (Woodard et al., 2014; Chailangkarn et al., 2016; Vessoni et al., 2016; Marchetto et al., 2017; Monzel et al., 2017). Those studies apply “Poisson surprise method” to detect and visualize “synchronous bursting” in spike raster plots. Interestingly, some of these publications also include calcium imaging data, and the presented calcium traces clearly show asynchronous calcium peaks and the absence of neuronal population wide synchronous activity (Chailangkarn et al., 2016; Monzel et al., 2017). As described recently, the “Poisson surprise method” for the detection of synchronous bursts has limitations such as their tendency to overestimate synchronous bursting in spike trains containing sparse or no synchronous bursting activity (Cotterill and Eglen, 2018).

By using a semi-automatic approach, we demonstrate the robust detection of population bursts and avoid spurious detection of population bursts by visual inspection for plausibility. Since the parameters spikes per minute and number or percentage of active electrodes do not allow to completely describe neuronal network properties, we used a population burst analysis tool box to describe occurrence and properties of neuronal network generated population bursts. The number of PB provides the information when in development population bursting starts in neuronal networks, however, it does not provide information, if the neuronal population is partially or highly synchronously active. In synchronously active human iPSC-derived 3D neural aggregate cultures, more than 50% of all recorded spikes are organized as population bursts, which is similar to highly synchronously active primary cortical neuronal networks *in vitro* [e.g., (Illes et al., 2014)]. Since “% spikes in PB” shows lower standard deviations than the Cohen’s kappa value and “% spikes in PB” is reflecting reliably the synchrony seen visually, we concluded that “% spikes in PB” represents a better parameter to describe the degree of synchrony in a neuronal network. We showed that the properties of population bursts of hiPSC neuronal networks change over time. The duration of population bursts and inter population burst interval show low standard deviations in comparison to the parameter PB amplitude, and both were suitable parameters to describe the similarities between neuronal network activity patterns recorded from different neuronal networks. Since 3D neuronal aggregate cultures show dynamic movement over time in culture (see Supplementary Figure S1), we surmise that the relatively high standard deviations of PB amplitude relies on the changing distance between bursting neurons and recording electrodes.

### Are the Time-Frames of Human and Rodent Functional Neuronal Circuit Development *in vitro* Different?

We demonstrated for the first time that human neuronal assemblies that are several hundred micrometers apart from each other, established a synchronously active neuronal network within 2 weeks of cultivation. Moreover, in more than 70 individual neural cultures from three different hiPSC lines, we observed that the transition from asynchronous into synchronous network activity occurred within 2 weeks in culture. These findings imply that human neuronal maturation processes, such as neurite outgrowth, synaptogenesis, spine development, ion-channel expression, and neurotransmitter system, essential for functional interconnection of neurons, occurred within less than 2 weeks *in vitro*. Indeed, by super-confocal imaging we confirmed that all these morphological properties (neurite outgrowth, vGlut1/PSD-95 synaptic proteins, dendritic spine development) were present in neurons within 3D-neural aggregate cultures (see Figure 6). Moreover, patch-clamp recordings revealed high electrophysiological functionality of neurons within 3D-neural aggregate cultures (see Figure 7). Our findings at the synaptic, single neuronal level and neuronal network level not only demonstrate that *in vitro* human functional neuronal circuit formation does not require several months (Amin et al., 2016; Fukushima et al., 2016; Odawara et al., 2016), but also that the time scale of human neuronal circuit formation *in vitro* is more similar to those of rodent neuronal cultures (Pine, 1980; Van Pelt et al., 2004; Illes et al., 2014). Thus, we do not find evidence that the time period of human and rodent neuronal circuit formation *in vitro* shows any species-specific differences.

### Future Translational Medical Application of Personalized Solely Human iPSC-Based *in vitro* Models

We demonstrated that combining purely hiPSC-derived neural cells with MEA recording devices allows for the generation of drug sensitive highly synchronously active human neurons which can be used to understand the impact of novel identified neuropharmacological drug candidates on human neuronal network level.

Since the human neuronal functional model presented here is solely comprised of cells obtained from a particular individual, it represents an approach for neurological diseases and neuropsychiatric disorder modeling superior to co-culture. For example, in bipolar disorder or schizophrenia, abnormalities for neuronal cells and astrocytes have been speculated (Halassa et al., 2007; Hercher et al., 2014; Ongur et al., 2014). Astrocytes play a key role in the synaptic communication and neuronal network function. Our present approach would allow person-specific functional neuronal modeling of bipolar disorder and schizophrenia, in which patient-specific neurons form a functional synchronously active network within patient-specific astroglial network.

To summarize, the presented approach of solely hiPSC-based functional neuronal networks paves the way for person-specific drug screening, neurological disease and neuropsychiatric disorder modeling.

## Materials and Methods

### Generation of iPSC Lines and Neural Induction

All lines were cultured under feeder-free conditions in Cellartis DEF-CS (Takara Bio Europe AB) at 37°C in a humidified atmosphere of 5% CO_2_ in air. Two controls (C1, C2) iPS cells (Vizlin-Hodzic et al., 2017) and a commercial iPS cell line, Cellartis DEF-hiPSC ChiPSC4, were used for neural induction by applying the DUAL-SMAD inhibition protocol (Shi et al., 2012a). The detailed neural differentiation procedure for iPSC lines is described in (Hayashi et al., 2015; Vizlin-Hodzic et al., 2017). Cryostock of hiPSC-NSC cultures were generated by passaging cells by using Accutase 20–24 days post neural induction in hiPSC cultures. Cell suspension of hiPSC-NSC were stored in 10%-DMSO solution and cryostocks were kept at −152°C.

### 3D-Neural Aggregate Formation and Neuronal Differentiation

Frozen stocks of hiPSC-NSC were thawed and cultured in neural culture media on PLO [0.01 mg/ml]/laminin [2 μg/ml] coated 3.5 cm culture plates. Neural culture media comprise of, DMEM/F12 GlutaMAX, Neurobasal, N2 supplement, B27 supplement, 5 μg ml^−1^ insulin, 1 mM Ultra glutamine, 100 μM non-essential amino acids, 100 μM 2-mercaptoethanol, 50 U ml^−1^ penicillin and streptomycin. After 7–10 days, 3D-neural aggregates with diameters ≤150 μM were manually transferred on PLO/laminin-coated coverslips or MEAs. For neuronal differentiation, BrainPhys-media supplemented with N2 supplement, B27 with vitamin A, 2 mM Ultra glutamine, 50 U ml^−1^ Pen/Strep, and 200 μM ascorbic acid were used. Half media exchanges were performed twice a week. Human BNDF, GNDF, NT-3, FGF8, TGF-β, (20 ng/ml each, all Peprotech) and DAPT (Tocris) were used as neurotrophic factors.

### Immunocytochemistry and Confocal Imaging

The procedure for immunocytochemistry is described in our previous study (Vizlin-Hodzic et al., 2017). Confocal imaging was performed by LSM 510 META or LSM 710 META (Zeiss). 5 μm optical slices were collected with confocal laser scanning microscopes to visualize NSC (Nestin, CD133, PAX-6), astrocytes (GFAP, S100b), oligodendrocytes (O4), cortical neuronal subtypes (Brn2A, Ctip2, SatB2), neurons (MAP-2AB), and proliferating cells (Ki67). Optical slices of 0.5 μm were collected with confocal laser scanning microscopes to visualize vGlut1, PSD-95 dot-like locations of synaptic markers and βTubIII^+^-spines.

### Cell-Attached and Whole-Cell Recordings

For electrophysiological experiments, 3D neural aggregates were generated as described before. 3D neural aggregates were passaged and plated on PLO/laminin-coated Ibidi μ-dishes (Ibidi) and maintained in BrainPhys culture media comprising supplements as described before. The μ-dishes were mounted under a differential interference microscope (Nikon E600FN) together with a CCD camera (Sony XC-73CE) to visually identify the cells and to visualize the recording electrode connected to the neuron via a borosilicate glass micropipette (resistance 3-6 MΩ). Cells were perfused (2–3 ml/min) with artificial CSF (ASCF) containing: 1 mM NaH_2_PO_4_, 123 mM NaCl, 26 mM NaHCO_3_, 3 mM KCl, 2 mM MgCl_2_, 1 mM CaCl_2_, and 10 mM D-glucose. The micropipette was filled with an intracellular solution containing; 127 mM K-gluconate, 8 mM KCl, 10 mM HEPES, 15 mM phosphocreatine, 4 mM Mg-ATP, 0.3 mM Na-GTP (pH ∼7.3 and osmolality 280–300 mOsm). Patch-clamp recordings were performed on cells at the edge of 3D-neural aggregates visually identified using infrared differential interference contrast video microscopy. The data was collected with a sampling frequency of 10 kHz and filtered at 3 kHz by an EPC-9 amplifier (HEKA Elektronik). After opening, the cell was allowed to rest for 5 min before recordings started. Series resistance was monitored using a 20 ms 10 mV hyperpolarizing pulse. The series resistance was not allowed to exceed 20 MΩ in whole-cell recordings, or to change more than 20% during an experiment, otherwise the experiment was discarded. Whole-cell recordings were carried out at 32°C.

Spontaneous current activity was recorded in cell-attached, spontaneous synaptic activity (i.e., EPSCs and IPSCs) was recorded in whole-cell voltage-clamp and the firing response to step-wise current injections (300 ms) was recorded in whole-cell current-clamp. For spontaneous synaptic activity cells were clamped at −70 mV for recordings of α-amino-3-hydroxy-5-methyl-4-Isoxazolepropionic acid receptor (AMPAR) mediated excitatory postsynaptic currents (EPSCs) and at 0 mV for recordings of γ-aminobutyric acid receptor (GABAR) mediated inhibitory postsynaptic currents (IPSCs).

All recordings have been performed between second (14 days) and third week (21 days) *in vitro*.

### Multi-Electrode Array Recordings and Pharmacological Experiments

Two to five hiPSC-3D-neural aggregates were seeded as a 5 μl drop directly on PLO/laminin coated electrode arrays of 6-well MEAs or one-well MEAs. After 1h, BrainPhys^TM^ media with supplements (described above) was added. Half media exchanges were performed twice a week. MEAs had a square grid of planar Ti/TiAu electrodes with PEDOT-CNT (carbon nanotube poly-3,4-ethylene-dioxythiophene) of 30 μm diameter and 200 μm spacing. Electrode configuration was nine recording electrodes per well in the 6-well MEAs and 60 electrodes per well in the standard MEAs. Recordings have been performed in BrainPhys media supplemented with B27, N2, and L-glutamine. For pharmacological experiments, cultivation media was removed and fresh BrainPhys media without any supplements was added (300 μl per well of 6-well MEA or 1 ml on one-well MEA). BrainPhys media was continuously bubbled with gas containing 95% O_2_ and 5% CO_2_. In DMSO-solved Picrotoxin, D-AP-5, CNQX, TTX and GABA (all Tocris) aliquots were solved in BrainPhys media before using. After application of drugs, at least 16 min of consecutive 2-min recordings were performed. MEA-electrodes had an input impedance of 30–50 kΩ according to the specifications of the manufacturer (Multi Channel Systems). The recording sampling rate 25 on all MEA electrodes. MC_Rack (Multi Channel Systems) was used to visualized and stored MEA data. Offline-spike detection was performed by the SPANNER software suite [RESULTS Medical; see also (Illes et al., 2014)]. Synchronous network activity was analyzed by population burst (PB) detection using custom-built Matlab software [see Figure 1 and (Hedrich et al., 2014)].

### Statistical Analysis

For statistical analysis of presented data in Figure 4, 8, we performed paired, two-sided t-tests of indicated groups using GraphPad prism (version 5). All presented data show mean value ± standard deviation (SD). Asterisks indicate significant values (^∗^*p* < 0.05, ^∗∗^*p* < 0.01, ^∗∗∗^*p* < 0.001, and ^∗∗∗∗^*p* < 0.0001).

## Ethics Statement

Work with human iPSC lines were approved by “regionala etikprövningsnämnden Göteborg, with the DNR 172-08.”

## Author Contributions

SI designed the study, wrote the manuscript, performed the experiments, data acquisition, analysis, and interpretation. JI performed the most of the experiments, data acquisition, analysis and interpretation, figure preparation, and manuscript writing. MA and HS performed patch clamp recording including data analysis and figure preparation. ST performed data analysis and revised the manuscript draft. DV-H performed some neural induction of human iPSC into NSC and NSC characterization and revised the manuscript. HÅ, KF, and EH performed the data interpretation and revised the manuscript draft.

## Conflict of Interest Statement

The authors declare that the research was conducted in the absence of any commercial or financial relationships that could be construed as a potential conflict of interest.

## Supplementary Material

FIGURE S1 Morphology of 3D neural aggregates cultured on 6-well MEAs over time. Phase contrast images show the morphology of three individual 3D neural aggregate cultures on 6-well MEAs over a time period of 4 weeks.

TABLE S1 Inter-culture comparison of neuronal network parameters. Table shows the individual values, mean, standard deviation and coefficient of variation of neuronal network parameters recorded and analyzed from 3D neuronal aggregate cultures presented in Figure 5A.
