# Supplementary figures and images for: Robust Generation of Person-Specific, Synchronously Active Neuronal Networks Using Purely Isogenic Human iPSC-3D Neural Aggregate Cultures
Source: Front Neurosci. 2019 Apr 24;13:351. doi: 10.3389/fnins.2019.00351 (PMC6491690; doi:10.3389/fnins.2019.00351)

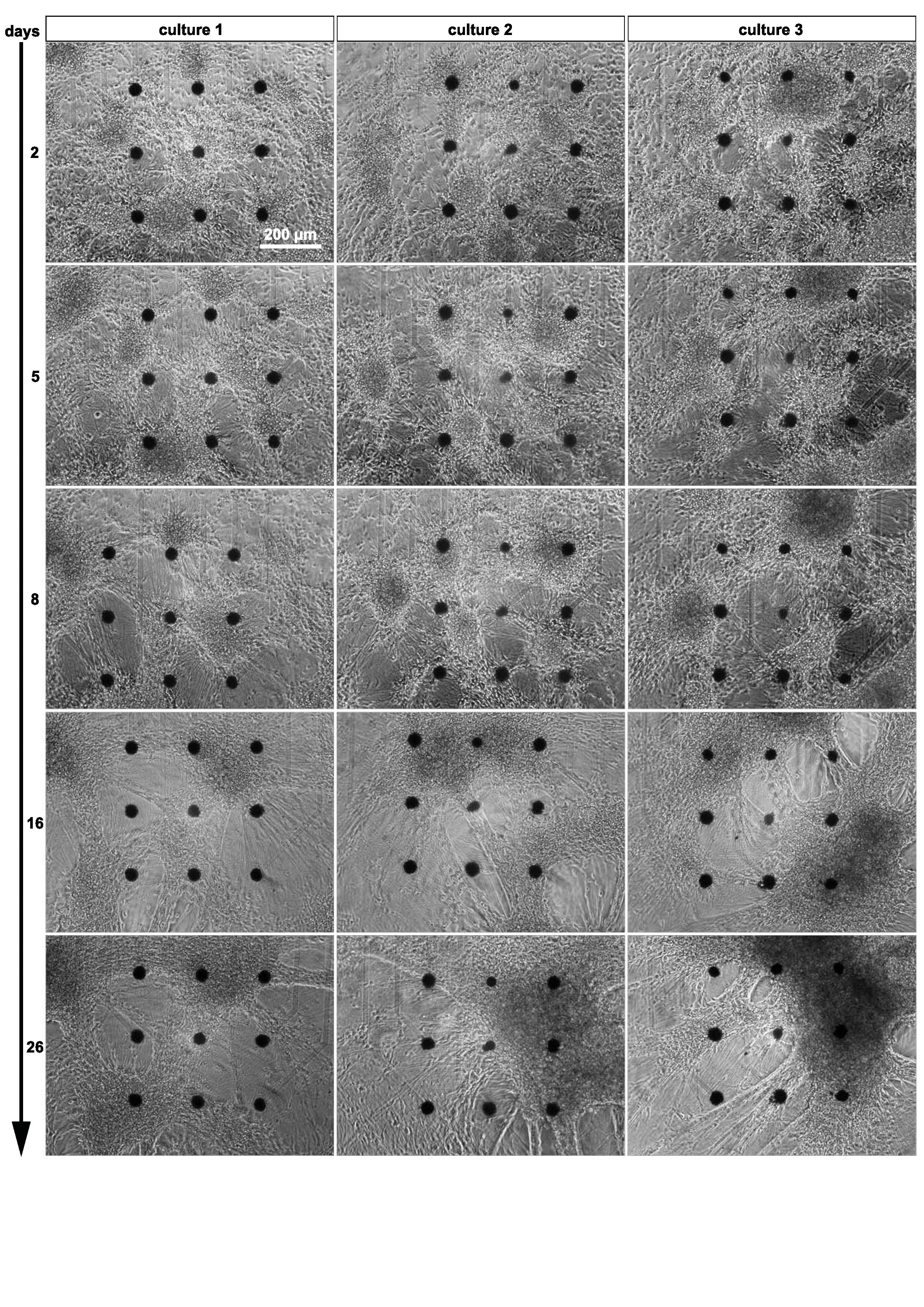

Supplement: FIGURE S1 — Morphology of 3D neural aggregates cultured on 6-well MEAs over time. Phase contrast images show the morphology of three individual 3D neural aggregate cultures on 6-well MEAs over a time period of 4 weeks. [file Image_1.JPEG]

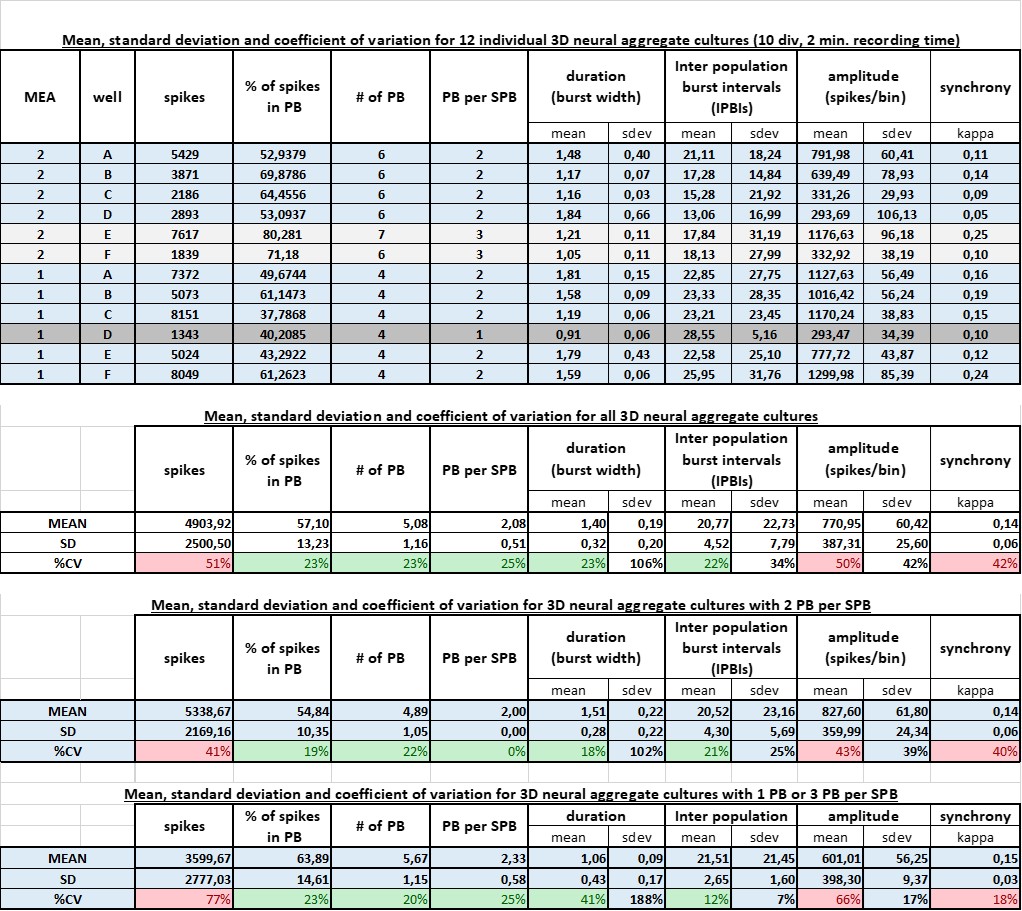

Supplement: TABLE S1 — Inter-culture comparison of neuronal network parameters. Table shows the individual values, mean, standard deviation and coefficient of variation of neuronal network parameters recorded and analyzed from 3D neuronal aggregate cultures presented in Figure 5A. [file Image_2.JPEG]
